# The Efficacy of Interlimb-Coordinated Intervention on Gait and Motor Function Recovery in Patients with Acute Stroke: A Multi-Center Randomized Controlled Trial Study Protocol

**DOI:** 10.3390/brainsci11111495

**Published:** 2021-11-12

**Authors:** Wai-Leung Ambrose Lo, Dandan Chen, Jiangli Zhao, Yan Leng, Ruihao Bian, Wenzhu Huang, Yahui Liang, Yu-Rong Mao, Dong-Feng Huang

**Affiliations:** 1 Guangdong Engineering and Technology Research Centre for Rehabilitation Medicine and Translation, Sun Yat-sen University, Guangzhou 510080, China; luowliang@mail.sysu.edu.cn (W.-L.A.L.); chend87@mail2.sysu.edu.cn (D.C.); zhaojl6@mail.sysu.edu.cn (J.Z.); lengyan@mail2.sysu.edu.cn (Y.L.); bianrh@mail2.sysu.edu.cn (R.B.); 2 Department of Rehabilitation Medicine, The First Affiliated Hospital, Sun Yat-sen University, Guangzhou 510080, China; 3 Department of Rehabilitation Medicine, The Seventh Affiliated Hospital, Sun Yat-sen University, Shenzhen 518132, China; 4 Department of Rehabilitation Medicine, The Fifth People’s Hospital of Foshan, Foshan 528211, China; xqhwz@mail2.sysu.edu.cn; 5 Department of Traditional Chinese Medicine, Beijing Rehabilitation Hospital of Capital Medical University, Beijing 100144, China; liangyahui@mail.ccmu.edu.cn

**Keywords:** arm cycling, interlimb coordinated, limb linkage, gait, stroke, EEG, MRI

## Abstract

Background: The efficacy of interlimb-coordinated training on gait and upper limb functional improvement remains unclear. The latest published randomized controlled trials have supported the potential benefits of interlimb-coordinated training to enhance gait function. Upper limb functional recovery may also benefit from interlimb-coordinated training since most everyday activities require the coordinated use of both hands to complete a task. This study investigates the efficacy of interlimb-coordinated training on gait and upper limb functional recovery over a short-medium term period. Methods: A total of 226 acute stroke patients will be recruited from four centres over four years. Patients will be randomly allocated to either conventional therapy or conventional therapy plus interlimb-coordinated training. Outcomes will be recorded at baseline, after 2 weeks of intervention, and at 3- and 6-months post-intervention. Gait speed is the primary outcome measure. Secondary outcome measures include Fugl–Meyer Assessment of Motor Recovery, Berg Balance Scale, Timed Up and Go test, Action Research Arm Test, electroencephalography, and magnetic resonance imaging. Conclusion: The results of this trial will provide an in-depth understanding of the efficacy of early interlimb-coordinated intervention on gait and upper functional rehabilitation and how it may relate to the neural plasticity process.

## 1. Introduction

Stroke is among the top-ranked cause of disability-adjusted life years worldwide [1]. It is the leading cause of disability in China [2], with approximately 3 million new stroke cases every year [3], making China the country with the highest prevalence of stroke in the world [4]. The Global Burden of Disease Study [5] stated that more effective interventions are needed. Gait and upper limb dysfunction are common among patients with stroke. Early literature indicated that almost two-thirds of the stroke survivors initially have mobility deficit and that over 30% of stroke survivors could not walk independently [6]. One year after stroke occurrence, half of the stroke survivors could not complete a 6 min walk test, and those who could were only able to achieve 40% of the predicted normal distance [7]. Only 5% to 20% of stroke survivors recovered their upper limb function completely, 25% of stroke survivors recovered part of upper limb function, and 60% of them lost upper limb function completely [8].

Effective gait recovery is essential to safely conduct daily living activities and to improve quality of life [9]. Gait speed is regarded as an indicator for quality of life and further stroke incidence [10]. The average gait speed of healthy individuals in the age group of 40–80 is between 1.26–1.41 m/s for men and 1.13–1.39 m/s [11] for women. Patients with subacute and chronic stroke were reported to have an average gait speed of 0.33 m/s [12] and 0.65 m/s [13] respectively. Despite advancements in rehabilitation technology, 90% of stroke survivors continue to experience mobility impairment [14]. Traditional gait intervention was conducted on the ground or on a treadmill with or without body weight support [15] in order to provide high-intensity, repetitive, task-specific training interventions to promote an improvement in gait function [16]. It has been reported that traditional gait intervention can achieve gait speed improvements of between 0.03 m/s [17] to 0.1 m/s [18]. The average minimal clinically important difference (MCID) for gait speed across patients with different stroke onsets was reported to be 0.175 m/s as the numerical parameter for a meaningful improvement in walking ability [19]. There is emerging evidence to suggest that gait recovery may remain suboptimal due to the fact that the paretic motor neuron pools are only partially activated by the nervous system, and the current gait rehabilitation regime may not be at a high enough intensity to activate these motor neuron pools [20]. Thus, this casts some doubts over the efficacy of traditional walking training in patients who have experienced stroke, and a new rehabilitation strategy is urgently needed. An increasing amount of evidence provides insight into the role of the upper limb, which may potentially play in gait recovery. A study on the effectiveness of stationary cycling training in chronic stroke patients demonstrated a significant improvement in gait [21]. It was suggested that the cycling motion shares a common locomotion pattern with walking based on the reciprocal lower limb muscles coordination [22]. The coordination of four-limb motion has been coined “interlimb coordination”, where all four limbs move in coordination to accomplish a task, and this has been recently proposed to enhance limb movement control through an increase of neural coupling between arms and legs [23]. Examples of interlimb-coordinated tasks are the use of a rowing machine, elliptical machine, and bicycle ergometer [24]. A recently published study that included healthy participants reported that upper limb muscles drive lower-limb muscle activity during a specific gait phase via the subcortical and cortical pathways to achieve intermuscular coherence between the upper and lower limbs [25]. This provides further evidence to support the role of upper limb in gait function and highlights the importance of upper and lower limb coordination to facilitate gait recovery. Rhythmic arm cycling training in patients with chronic stroke was reported to promote a significant improvement in gait, balance, lower limb motor function, and enhanced activity in the dorsiflexion muscle during the swing phase of walking [26]. The most recently published preliminary randomized controlled trial investigated the effect of the interlimb coordinated protocol in patients with chronic stroke [24]. A range of interlimb coordinated tasks were provided to chronic stroke patients for 2 months (3 h per week) in an outpatient setting. The results showed that the interlimb-coordinated training group had significantly higher improvement in their Fugl–Meyer Assessment scores, Rivermead Visual Gait Assessment, and modified Rankin Scale than the control group, providing some support for the potential role of the upper limbs in gait function recovery and the potential benefits of interlimb-coordinated intervention [27]. However, the study that adopted interlimb-coordinated intervention was a preliminary trial without adequately powered sample calculation, increasing the likelihood of a type II error. In addition, patients with chronic stroke were recruited in the study, and it remains unclear as to whether interlimb-coordinated intervention could be adopted during the acute phase. The neural plasticity changes induced by interlimb-coordinated intervention are unknown as no studies have investigated the changes at a cortical level. However, studies conducted in healthy individuals showed stronger activations of the cortex supplementary motor area (SMA), premotor area [28], and cerebellum [29] during ipsilateral arm and leg movement in the opposite direction than during ipsilateral arm and leg movement in the same directions and during single-limb movement. Thus, it is logical to expect that stroke patients who have undergone interlimb coordinated intervention may have stronger cortical activation that correlates with an improvement in gait function. To date, only one study has been found that investigated the neural integrity at the spinal level induced by arm cycling [26], which confirmed its modulation effect on gait and balance in chronic stroke patients. The neuroplasticity at the cortical level induced by interlimb-coordinated intervention remains unclear. It is also unknown if interlimb-coordinated intervention may yield that is in comparable clinical benefits to those observed in the chronic stroke phases when provided at the acute stages.

Upper limb functional recovery in order to perform fine motions, such as grasping, finger pinching, and individual finger dexterity, continues to be a challenge for stroke survivors [30]. The majority of everyday activities require the coordinated use of both hands [31]. It is common that one of the upper limb stabilizes a trunk or an object itself in order to allow manipulation by the other limb, or both of the upper limbs may be needed to manipulate the object for a task to be completed [24]. The impairment of bimanual coordination in patients with stroke have been reported in several studies, and impairments in coordination are not related to the lateral deviation of the impaired limb [32,33]. A large scale systematic review attempted to identify which type of intervention promotes arm and hand functional recovery post stroke, and the authors reported that no high-quality evidence was available to support any of the interventions that are currently used as part of routine practice [34]. The majority of the published literature investigated the efficiency of single-limb rehabilitation regimes, such as a single-armed robotic device [35], constraint-induced movement therapy [36], or commercial gaming devices such as the Microsoft Xbox [37]. While these intervention regimes demonstrated various degrees of success in upper limb functional recovery and cortical reorganization, the general results of upper limb function recovery remain unsatisfactory, as evidenced by the fact that a large proportion of patients with stroke continues to have an upper limb functional deficit years after stroke occurrence. Thus, patients with stroke may theoretically benefit more from bimanual training tasks than unimanual tasks. A preliminary study that compared the benefits between single-limb training with double-limb robotic devices reported results favouring double-limb training [27]. To date, there is also insufficient evidence to demonstrate whether interlimb-coordinated intervention is beneficial in upper limb function recovery when it is provided at the acute stage. 

The primary aim of the present study is to investigate the effect of interlimb-coordinated intervention on gait and motor function in patients with stroke at the acute stage. It is hypothesized that acute stroke patients who receive interlimb-coordinated intervention will significantly improve gait and upper-limb motor function. These improvements would be significantly higher than those who receive conventional therapy immediately after the 2 weeks of intervention and at the 3- and 6-month follow-up periods. The secondary aim is to investigate the neural plasticity changes that are induced by interlimb coordinated intervention after 2 weeks of intervention and at the 3- and 6-month follow-up periods. It is also hypothesized that the participants who undergo interlimb-coordinated intervention would have stronger cortical activation at rest and during gait and a shorter neural processing time to initiate a motor task than the participants in the control group immediately after intervention and at the 3- and 6-month follow-up periods. 

## 2. Materials and Methods 

### 2.1. Study Design

This study is a parallel, single-blinded, multi-center randomized controlled trial to evaluate the effect of interlimb-coordinated intervention on gait and motor function recovery in patients with acute stroke. 

### 2.2. Study Setting

**Figure 1:**
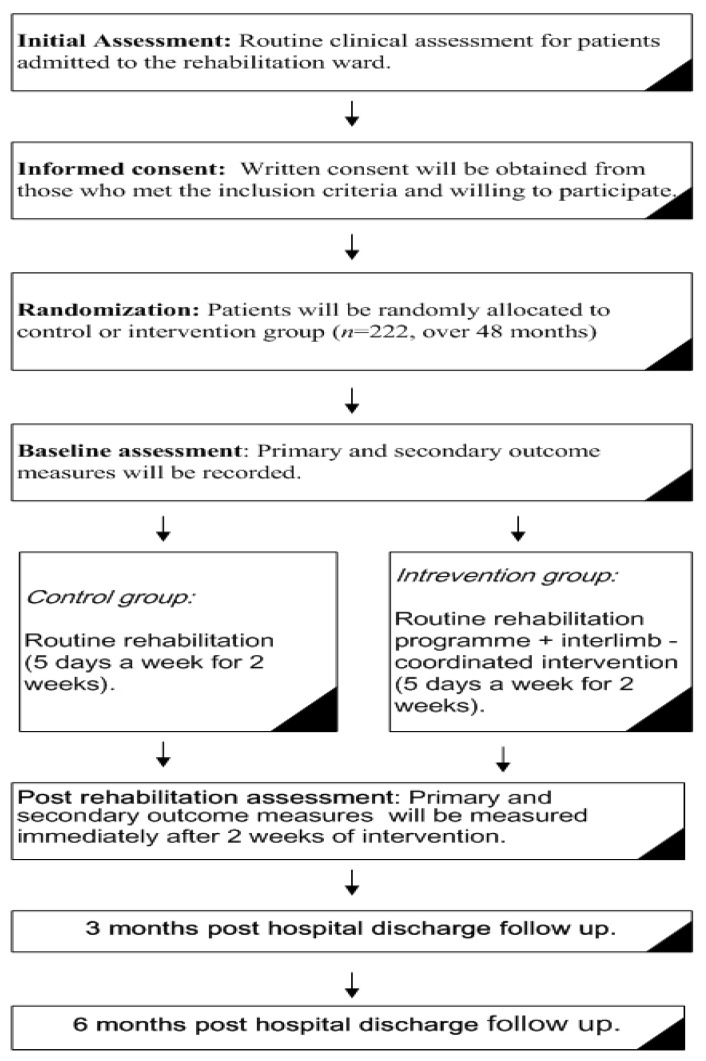
Flow diagram of the study procedure.

Data collection will take place at the rehabilitation department of four tertiary hospitals located in different regions of China: (1) The First Affiliated Hospital of Sun Yat-Sen University; (2) The Seventh Affiliated Hospital of Sun Yat-sen University; (3) The Fifth People's Hospital of Foshan Institute of Science and Technology; and (4) the Beijing Rehabilitation Hospital of Capital Medical University. Participants will be recruited from the inpatient wards of the participating centers. The data collection process is expected to last for 4 years. All interventions will be delivered by trained healthcare professionals within these centers. Figure 1 shows the study procedure. 

### 2.3. Recruitment

Suitable participants will be identified from patients who are admitted to the rehabilitation department due to stroke. The initial screen for eligibility will be conducted as part of the routine clinical assessment by members of the medical team. Written information about the study will first be provided to all potentially suitable participants before being approached by the research team to inquire as to whether they are interested in participating in the study. Written consent will be obtained from participants who express willingness to participate. All of the non-recruited patients and the reasons for exclusion will be recorded in a screening log. 

### 2.4. Sample Population 

The inclusion criteria for the stroke group are as follows: (1) the first occurrence of stroke with hemiparesis within the first month as confirmed by computed tomography or magnetic resonance imaging (MRI); (2) participants must be aged between 40- to 79-years-old; (3) Brunnstrom stage 3; (4) modified Ashworth scale score ≤2; (5) medically stable and able to sit unsupported for at least 30 min; (6) able to walk 10 m with or without walking aid; (7) no severe cognitive impairment, as defined by the Mini-mental State Examination (scale score greater than 10) [38], and (8) at least five degrees of active wrist extension and ankle dorsiflexion are available. The exclusion criteria are as follows: (1) comorbidity conditions including congestive heart failure, deep vein thrombosis, and malignant progressive hypertension; (2) lower limb fracture; (3) history of mental illness or on anti-psychotic drugs. The rationale to include only participants who could sit unsupported for 30 min was due to that fact that interlimb-coordinated interventions are conducted in a seated position. The Brunnstrom stage of recovery is included as a criterion to ensure that the participants have recovered some of the motor function before participation in the intervention program. The modified Ashworth scale is to ensure that motor function is not affected by high level of muscle spasticity.

### 2.5. Randomization

Eligible participants will be randomly assigned to either the experimental or the control group at a ratio of 1:1 without stratification. A statistical expert from the Faculty of Medical Statistics and Epidemiology, Sun Yat-sen University, computed the random allocation sequence. The sequence will be uploaded onto a central operating system. Research team members will access the system at the time of participant enrolment by dialing the central phone number to obtain the allocation. The operating system will provide the numbers of 001 and 002, which correspond to the intervention group and control group, respectively. Each participant will be assigned a unique identification number to maintain confidentiality. 

### 2.6. Ethics

Ethical approval was obtained from the Medical Ethical Committee of the First Affiliated Hospital of Sun Yat-Sen University (Ethics Number: [2020]430). The clinical trial is registered with the Chinese Clinical Trial Registry (Registration Number: ChiCTR2000040137, prospective registration on the 22 November 2020). All relevant parties will be informed of any important modifications to protocol modifications. All of the participants who are invited to participant in the study will be given time to consider if they wish to take part in the trial and will be encouraged to ask any questions. All of the participants will be free to withdraw from the trial at any time without providing a reason. The participants will be able to withdraw themselves completely, in which case, all of the collected data will be excluded from the study, or to withdraw only from further assessment or intervention, in which case, all of the collected data will be included in the final analysis depending on personal preference. Medical expenses that are incurred as part of the research are covered by the research grant. Travel reimbursement will also be provided to all participants during the follow-up periods to promote a strong adherence rate. 

### 2.7. Outcome Measures

#### 2.7.1. Primary Outcome Measure

The primary outcome measure is gait speed, which will be assessed by the 10 m walk test. 

#### 2.7.2. Secondary Outcome Measure

All outcome measures will be recorded at baseline, post-intervention, and at 3 and 6 months post-intervention. Table 1 presents a brief description of each of the secondary outcome measures that will be recorded at all participating centres. The outcome measures of the 10 m walk test, Fugl–Meyer Assessment, and Action Research Arm Test are the recommended outcome measures by expert consensus from national societies in the United States for body function and activity [39]. The Berg Balance Scale (BBS) was also reported to be a tool that is valid, reliable, and sensitive to change in post-stroke balance impairment assessments despite the potential concern of the ceiling and flooring effect [40]. The timed-up and go test (TUG) was initially developed to identify balance impairment [41] and has been reported as a reliable, valid, and easy-to-administer clinical tool that can be used to assess mobility and balance [42]. Both the BBS [43] and TUG [44] are strong fall predictors. The Instrumental Activities of Daily Living has also been reported as an international gold standard despite its limitations and biases [45].

### 2.8. Event Related Potentials 

**Figure 2:**
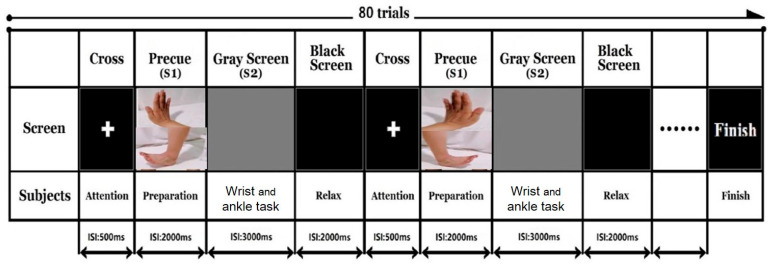
Event related potential paradigm to measure contingent negative variation.

The event-related potential (ERP) waveform contingent negative variation (CNV) will be recorded by a 32-channel QuickAmp amplifier and Ag/AgCl scalp electrodes (BrainProducts, Gilching, Germany). CNV is a sustained negativity that occurs after an initial stimulus (S1) and before the imperative stimulus (S2) that requires a response [51]. It is a waveform that occurs during the preparatory period between the cue and response stimuli and is an indicator for motor task initiation [52], including hand movement and gait tasks. The electrodes will be positioned in accordance with the international 10–20 system, with the additional electrodes within the covered area. The following electrodes will be used: FP1, FP2, F7, F3, Fz, F4, F8, T7, C3, Cz, C4, T8, P7, P3, P4, P8, O1, O2, TP9, TP10, FC5, FC1, FC2, FC6, CP5, CP1, CP2, CP6, PO9, O1, O2, PO10; FCz will be used as a reference electrode, and AFz will be used as a ground electrode. Data will be recorded with a sample rate of 1000 Hz in direct current mode. Each electrode will be filled with conductive gel to maintain impedance below 5 kΩ. Alpha, beta, gamma, and theta wave oscillation will be recorded and analysed at resting state. The ERP paradigm is illustrated in Figure 2.

### 2.9. fMRI

Data acquisition of functional magnetic resonance imaging (fMRI) will follow the scanning procedure from a previous study where gait function improvement was assessed [53]. The T1-weighted data set of the entire brain will be acquired for each participant. The scan parameters for blood oxygen level-dependent (BOLD)-weighted scans are as follows: TR = 200 ms, TE = 25 ms, field of view 200 × 200, matrix 64 × 64, and a slice thickness of 3 mm. The scanning paradigm involves five active movement blocks of ankle plantar flexion. Each block is triggered by an auditory command with a 20 s epoch of rest in between each block. The scanning time of the movement of the affected ankle is 200 s (the amount of time it takes to complete five active movement blocks of ankle plantar flexion). A metronome with the beat set to 30 beats per minute will be provided to set the pace of the ankle movement. All of the participants will be given sufficient time to practice the movement task prior to data recording. Imaging data will be analysed by the software Statistical Parametric Mapping 8 implemented in MATLAB (software available at https://www.fil.ion.ucl.ac.uk/spm/, accessed on the 9 November 2021). The anatomic space template was adopted to normalize the functional scan images. The within-subject before and after intervention will be analysed by paired sample *t*-test. 

### 2.10. fNIRS

The cortical activation level during gait will be recorded by a portable multi-channel frequency domain fNIRS system (BRITE, Artinis Medical Systems, Gelderland, The Netherlands). The particular regions of interest are the motor cortex (M1), supplementary motor cortex (SMC), and premotor cortex (PMC). The system consists of 28 optodes and is made up from 12 light source fibers and 16 detector fibers, giving 42 recording channels. The optode distance will be set at 3.0 cm and will be located over the bilateral fronto-parital cortices, including the primary supplementary, premotor cortices, prefrontal cortices, and sensorimotor area. The changes in the oxygenated hemoglobin concentration level will be recorded at rest and during 30 s of walking for five repetitions. 

### 2.11. Outcome Assessments

Each centre has a dedicated multidisciplinary assessment team who are specifically trained to deliver the outcome assessments. Team members consist of occupational therapists, physiotherapists, physicians, and nurses who will be blinded to the group allocation. 

### 2.12. Sample Size

Sample size calculation will be based on the primary outcome measures of gait speed. An increase in gait speed of 0.16 m/s has been stated as the minimal clinically significant difference in the first 60 days of stroke occurrence [54] that correlating with a meaningful improvement in disability level post intervention. The study will consider 0.3 m/s for power calculation since other study-reported changes below this value are likely to be measurement errors [55].

The sample size was determined using the sample size calculator G*Power. The calculation model is based on the F test family, the statistical “ANOVA: Repeated measures, between factors” test and the “A priori: Compute required sample size–given alpha power, and the effect size” as the power analysis type. Power calculation was based on a mean difference of 0.3 (mean of group 1 = 0.69; mean of group 2 = 0.39) and the standard deviance of each group of 0.75 with a total sample population of 60. A total of 206 participants (103 in each group) is required to provide 95% power and an effect size of 0.2. Taking a 10% missing rate into consideration, a final sample size of 226 participants (113 in each group) was determined. 

## 3. Procedure

The Standard Protocol Items: Recommendations of Interventional Trials (SPRIT) figure illustrates the overview of the schedule of all events of the study (Table 2). 

### 3.1. Control Group 

The Participants in the control group will receive conventional rehabilitation treatment and routine medical care. Conventional rehabilitation programs include physiotherapy and occupational therapy. The program includes muscle strengthening exercises, treadmill or over ground gait training exercise, balance training such as body weight transfer, upper limb training such as passive range of movement and foam rolling, and functional practice of daily living activities such as object transfer, peg hole exercises and object manipulation, and neuromuscular electrical stimulation. Rehabilitation will be provided 5 days a week for 2 weeks. The daily duration of all of the rehabilitation components will last for a total of 2 h. Table 3 presents the time allocation for each training item.

### 3.2. Intervention Group

**Figure 3:**
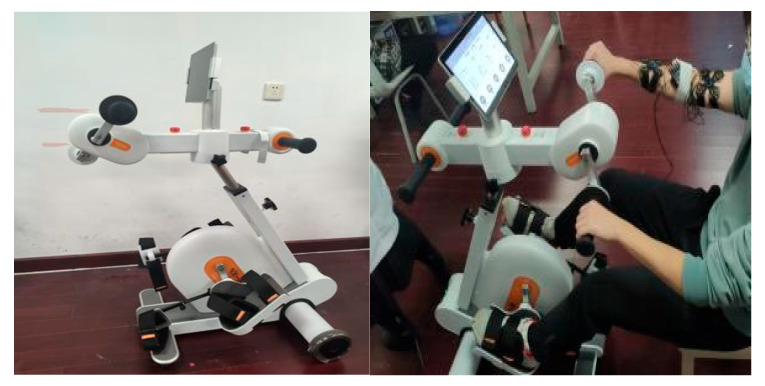
Bicycle ergometer for interlimb coordinated intervention.

The intervention group will receive interlimb-coordinated training for 20 min in addition to conventional training. Each of the conventional rehabilitation components will be shortened to reduce the overall conventional therapy program duration for a total of 20 min in the intervention group. This is to give equivalent therapeutic time among the two groups. Interlimb-coordinated training will be conducted on a bespoke bi-cycle ergometer where participants will be asked to perform arm and leg cycling simultaneously. A diagram of the bespoke bi-cycle ergometer is illustrated in Figure 3. The 20 min of interlimb-coordinated training will be divided into 10 min of active cycling and 10 min of active-assisted cycling. In active-assisted mode, the participants will be asked to primarily lead the movement with the paretic side, and assistance will be provided by the ergometer’s motor, which reduces the effort required from the participant. The bespoke bi-cycle ergometer is designed to have a separate motor on the left and right sides to ensure that assistance is only provided to the paretic side. In active cycling mode, the four motors that power the handlebars and pedals are linked to enable a synchronized reciprocal cycling motion of the upper and lower limbs simultaneously. Participants will be asked to cycle at a work rate that would provide a score of between 10–13 (light–somewhat hard) on the Borg’s Rating of Perceived Exertion scale [56]. The exact work rate on the cycle ergometer could not be pre-determined or fixed for every participant, as each participant is likely to have a different exercise capacity. Thus, a subjective Borg scale was adopted as an index to monitor work rate intensity. 

## 4. Safety and Adverse Event Reporting

Patients with acute stroke have a likelihood of morbidity and mortality. Procedures are in place to minimize the occurrence of adverse events. Medical doctors will assess participants to ensure safe rehabilitation participation. Medically unstable patients will not be recruited for the study. Participants will be asked to exercise according to their physical capability. Trained physiotherapists/occupational therapists who work closely with the inpatient clinical team will be onsite during the exercise sessions. Any issues raised during the exercise session will be reported to the inpatient medical team. The following safety reporting procedures are in place:

1. Serious adverse events (SAEs) are defined as an event that incurs harm to the participants, which may or may not require medical or surgical intervention as a preventative measure to avoid the following outcomes: death, further impairment of body function, damage to a body structure, and prolonged the hospitalization period.
2. All SAEs related to the interventions will be recorded on a SAEs event report form in accordance with the procedures of each research centre.
3. All of the SAEs related to the interventions must be reported to the Principal Investigator within 24 h of learning of the event.
4. SAEs that are related to the interventions will be reported to the University Clinical Trial Unit and Research Ethics Committee within 15 days of the occurrence of the SAEs. The following information will be reported to relevant parties: (1) the concerned research protocol; (2) a report on the description of the SAE and subsequent outcome; (3) a proposal of changes in response to the SAE to prevent further occurrence of SAEs

### 4.1. Data Management

Data will be recorded on the case report form at each site. A bespoke online data storage system will be developed for the central processing of the recorded data. Data entry will be conducted by at least two assessors to minimize data entry errors. Study conduct monitoring will be performed by a dedicated committee from the participating site on an annual basis. Recorded data will only be accessible by authorized personnel.

### 4.2. Data Analysis

All statistical analyses will be performed in IBM SPSS version 21 software (SPSS Inc., Chicago, IL, USA). Descriptive statistics will be conducted to describe the sample population. The demographic characteristics and baseline results for the primary and secondary outcome measures will be analyzed using analysis of variance (ANOVA) or Chi-square test for categorical data. A mixed-effect model analysis will be adopted to compare changes in all of the outcome measures between groups at the completion of the intervention and at the 3- and 6-month follow-ups. The mixed-effect model analysis will include treatment, time, and centre as fixed effects, and the participants will be included as the random effect. Changes in the outcome measures will be adjusted for age, sex, centre, and time. The level of statistical significance will be set at *p* < 0.05. Cohen’s d will be calculated to measure the effect size of the difference for every comparison. The effect size will be interpreted as follows: 0.2—small effect size; 0.5—medium effect size; and 0.8—large effect size. Stratification analysis will be conducted to explore the potential effect difference between centres. There are no planned interim analyses or stopping rules, as the power calculation has accounted for loss to follow-up. In the event of missing data during the follow-up period, the modified intention to treat principle will be followed with the last observation carried forward method. 

## 5. Discussion

Several clinical guidelines propose that active rehabilitation is to be provided within 24–48 h of stroke occurrence [57], as the most considerable amount of the recovery was reported to take place in the first month following stroke [58]. Available evidence supports the benefits of early rehabilitation intervention on motor recovery, but convincing evidence for an early interlimb coordinated intervention is lacking. Thus, this study will provide evidence on the feasibility and efficacy of interlimb coordinated movement as part of an early intervention program for gait and motor function improvement. The medium long-term efficacy of the interlimb coordinated intervention will also be assessed in this study. Several Cochrane reviews regarding the efficacy of other interventions on motor function recovery seldom report data beyond the initial discharge period [59] or has followed up beyond the three [60] or six month periods [61]. As motor function capacities evolve the most during the first month of stroke onset [62], it is anticipated that intervention provided during the acute stage of stroke has better clinical outcomes when compared to interventions that are provided at later stroke stages. Therefore, 3- and 6-month post-intervention follow-up data will increase the understanding of whether interlimb coordinated intervention is superior to conventional therapy in the longer term. 

The key principle of motor function recovery post stroke is neural plasticity. A previous study indicated that function improvement is related to cortical reorganization post-intervention [53]. However, the optimal strategy for intervention application in order to increase cortical activation and motor skill learning has yet to be determined [27]. Early literature indicated that the activation patterns related to bimanual task performance were not similar to unimanual tasks such as finger opposition [63]. Recent literature propagates the theory that unimanual motor tasks involve bi-hemispheric activation patterns that are similar to the bilateral neural activation that is typically observed during bimanual movements [64]. This theory is given further support by a study that reported bimanual coupling effects observed during affected and unaffected hand tasks in an individual with stroke [65]. Another study utilized fMRI to compare the inter-regional connectivity and inter-regional activation between the bimanual in-phase and bimanual antiphase upper limb task in healthy individuals. Significant activation of the supplementary motor area, cerebellum, thalamus, and the cingulate motor area were observed during the bimanual antiphase task but not during in-phase movement [66]. The connectivity analysis also indicated stronger neural coupling during antiphase movement than during bilateral in phase and unimanual movement. Antiphase movement is similar to gait and requires the precise spatial and temporal control of the limbs. Spatial and temporal control is also required during single-hand movement due to the inhibition of the ipsilateral motor area, as evidenced by the presence of neural activity in the bilateral motor cortex [67]. It is anticipated that the interlimb-coordinated intervention that is provided at the acute stage will provide higher inter-regional connectivity and cortical activation when compared to conventional therapy, providing further evidence to support the regime to improve motor function in patients with stroke. In conclusion, this proposed research is set to provide information on the interlimb coordinated intervention as an early intervention program for patients with stroke. 

## 6. Conclusions

The results of this trial will provide an in-depth understanding of the efficacy of early interlimb-coordinated intervention on gait and upper functional rehabilitation and how it may relate to the neural plasticity process.

## Figures and Tables

**Table 1:** A brief description of each of the secondary outcome measure that will be recorded at all participating centres.

Fugl-Meyer Assessment of Motor Recovery (FMA) [46]	It is a validated tool to evaluate motor function, balance, and joint function in stroke-related hemiplegic patients. The interpretation of FMA is as follows: <50 = Severe; 50–84 = Marked, 85–94 = Moderate; 95–99 = Slight.
Berg Balance scale (BBS) [47]	The BBS consists of 14 balance-related tasks that include sit to stand, stand to sit, and standing on one foot. It was developed to assess the ability to maintain dynamic and static balance.
Timed up and go test (TUG)	The test requires the person to rise from a chair and walk 3 m at a comfortable pace, turn around at the 3 m mark, and walk back to the starting point. The test’s score is the time it takes the person to complete the test. A cut off of 14 s was proposed to be “normal” [48].
Action Research Arm Test (ARAT) [49]	ARAT is a 19-item test that is divided into four sub-tests, which consist of grasp, grip, pinch, and gross arm movement. A score of between 0 to 4 is given to each task: 0—can perform none of the test; 1—performs part of the test; 2—takes abnormally long or has great difficulty in completing the test; 3—able to perform the test normally.
Instrumental Activities of Daily Living (IADL) [50]	The IADL [26] is a functional disability scale that assesses the functional level by asking whether a person receives personal help with daily living activities, such as using the telephone, getting to places outside the house, grocery shopping, preparing meals, doing housework or handyman work, laundry, taking medications, and managing finances.
Electroencephalography (EEG)/Event related potential (ERP)	Parameters of contingent negative variation (CNV), alpha, beta, gamma, and theta wave oscillation.
Functional magnetic resonance imaging (fMRI)	The voxel count T1-weighted data set of the entire brain will be acquired for each participant. The scan parameters for blood oxygen level-dependent weighted scans are as follows: TR = 200 ms, TE = 25 ms, field of view 200 × 200, matrix 64 × 64, and a slice thickness of 3 mm.
Functional Near-Infared Spectroscopy (fNIRS)	The optical system used in the study will be a multi-channel frequency domain NIRS system (BRITE, Artinis Medical Systems, Gelderland, The Netherlands). The particular regions of interest is motor cortex (M1), supplementary motor cortex (SMC), and premotor cortex (PMC).

**Table 2:** SPIRIT diagram for the study.

	Enrolment	Allocation	Post-Allocation			
Timepoint	0	0	Baseline	3 Weeks Intervention	3 Months	6 Months
**Enrolment:**						
Eligibility screen	X					
Informed consent	X					
**Allocation**		X				
**Interventions:**						
Routine with Interlimb coordinated			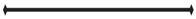			
Routine rehabilitation			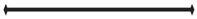			
**Assessments:**						
Gait speed			X	X	X	X
EEG			X	X	X	X
fNIRS			X	X	X	X
MRI			X	X	X	X
BBS			X	X	X	X
ARAT			X	X	X	X
FMA-UL			X	X	X	X
IADL			X	X	X	X

**Table 3:** Time allocation for each treatment in the control and intervention groups.

	Time (Minutes)	
Treatment Type	Control	Intervention
Muscle strengthening	20	17
Treadmill gait training	20	17
Balance training	20	17
Passive exercise	10	7
Upper limb training	20	17
Functional practice	20	17
Neuromuscular electrical stimulation	10	8
Interlimb coordinated	0	20
Total	120	120

## Data Availability

Not applicable.
